# The evolution and reconstruction of digital addiction: from compulsive consumption in the internet era to symbiotic dependence in the artificial intelligence era

**DOI:** 10.3389/fpsyg.2026.1858405

**Published:** 2026-07-27

**Authors:** Tianyu Jiang, Yonghe Wang, Caiyu Wang, Zan Zhou

**Affiliations:** 1 School of Physical Education, Shaoxing University, Shaoxing, China; 2 School of Education and Psychology, Shaoxing University, Shaoxing, China

**Keywords:** anthropomorphic interaction, artificial intelligence, cognitive offloading, digital addiction, intelligent symbiosis

## Abstract

With the development of generative artificial intelligence (AI), human-computer interaction has shifted from a primarily instrumental function to a more human-like symbiotic relationship. However, current research on digital addiction (DA) remains limited to the compulsive consumption characteristic of the Web 2.0 era and does not adequately address the new pathological features emerging with AI. This study uses a narrative review method to systematically trace the evolution from internet addiction disorder to smartphone addiction, and subsequently to AI addiction. Research indicates that DA has progressed from dopamine-driven sensory pursuits to anthropomorphic interaction and cognitive offloading, now influenced by oxytocin and the law of cognitive economy. Building on these findings, this article introduces the concept of “intelligent symbiotic digital addiction,” which encompasses “algorithmic intimacy disorder” at the emotional level and “generative dependency syndrome” at the cognitive level. Additionally, it presents the “dual-track drive” pathological theory of cognitive and emotional symbiosis, analyzes the internal mechanisms of subjectivity alienation, offers new perspectives for addressing pathological challenges in the AI era, and suggests directions for future algorithm ethics oversight and clinical intervention.

## 1 Introduction

With the development of the Internet, big data algorithms, and generative artificial intelligence (GenAI), digital technology has shifted from serving merely as a tool to becoming an essential instrument on which people depend. While individuals benefit from the convenience provided by digital technology, they also experience unprecedented uncertainty and a sense of loneliness. Consequently, digital devices have increasingly become psychological anchors for obtaining ontological security (Padgett, 2007). This profound psychological and functional dependence is progressively evolving into a social pathological phenomenon known as digital addiction (DA).

DA has evolved alongside recent developments in AI. Internet addiction disorder (IAD) in the Web 1.0 era and smartphone addiction in the Web 2.0 era primarily focus on users’ lack of self-control over content such as games and short videos (Widyanto and Griffiths, 2006; Young, 1998), while overlooking the “anthropomorphic interaction” and “intention understanding” characteristics of GenAI (Brandtzæg et al., 2021). The object of addiction has shifted from the original “addictive object” to the current “anthropomorphic subject.” These changes in content and interaction forms require more in-depth exploration.

Therefore, this study aims to clarify the evolutionary process of DA from the Internet era to the AI era, analyze the underlying mechanism of addictive motivation which has shifted from “content-driven” to “interaction-driven,” and propose a new concept of DA in the context of the AI era.

## 2 Methods

Although this study primarily employs a narrative review approach to analyze and reinterpret the evolution mechanism of DA, we also implemented a rigorous, transparent, and systematic literature search strategy. This approach was intended to minimize subjective selection bias and ensure an objective and comprehensive presentation of the research trajectory across Web 1.0, Web 2.0, and AI eras.

### 2.1 Search strategy and data sources

To capture a comprehensive multidisciplinary perspective, we conducted a systematic literature search across multiple authoritative databases, including WoS, Scopus, PubMed, and PsycINFO. The search strategy utilized was a customized Boolean logic combination to track the transformation of the human-computer interaction paradigm.

Web 1.0 era (device and network dependence): Search terms included (“Internet addiction disorder” or “IAD” or “compulsive internet use”) and (“impulse control” or “dopamine reward”).

Web 2.0 era (dependence on applications and algorithms): Search terms included (“smartphone addiction” or “algorithm addiction” or “social media dependence”) and (“algorithm capture” or “fear of missing out” or “FoMO”).

The Era of Generative AI (Cooperative Dependency): Search terms included (“Generative AI” or “ChatGPT” or “Large Language Model”) and (“Cognitive Offloading” or “Personification Interaction” or “AI Dependency” or “Human-Machine Coexistence”).

### 2.2 Inclusion and exclusion criteria

To ensure rigor in the analysis, strict inclusion and exclusion criteria were established. Inclusion criteria were as follows: (1) peer-reviewed empirical studies, theoretical frameworks, and high-quality systematic reviews; (2) studies that clearly describe the psychological, emotional, or cognitive mechanisms underlying the use of the analytical techniques; and (3) articles published in English. Exclusion criteria were: (1) preprints that have not undergone peer review, conference abstracts, or unsupported opinion articles; (2) studies that focus solely on technical design, code, or algorithm architecture of AI without addressing user psychology or behavioral governance; and (3) literature lacking clear empirical data or a solid theoretical foundation.

### 2.3 Data extraction and theoretical mapping

All retrieved literature was centrally managed, and duplicates were removed. Subsequently, we screened the titles and abstracts according to the pre-defined eligibility criteria to assess their relevance. It is important to emphasize that the concept reconfiguration and three-layer evolution framework proposed in this paper are not pre-defined templates designed to selectively include supportive articles. Rather, we applied the principle of theoretical saturation, which results from an exhaustive evaluation of literature across multiple databases. Specifically, tracking early clinical definitions and hardware obsession systematically supported the first layer; empirical studies on algorithm recommendation and psychological compensation formed the basis of the second layer; and the frontier paradigm exploring human-machine cognitive integration provided the foundation for the third layer. This two-way alignment ensured that our proposed framework is not only comprehensively supported by empirical evidence but also possesses strong explanatory power, while maintaining narrative depth and effectively avoiding selection bias.

## 3 Evolution of the concept of digital addiction

With the advancement of digital technology, the concept of DA has undergone fundamental changes. Academic research has shifted its focus from compulsive consumption during the Web 1.0 era, to psychological compensation under algorithmic manipulation in the Web 2.0 era, and now to interactive addiction triggered by GenAI. This progression illustrates how the addictive object has evolved from a “passive tool” to an “anthropomorphic subject,” highlighting the need to re-examine the boundaries of human-machine interaction.

Since the concept of IAD was proposed by Goldberg (1996) and established by Young (1998), the academic community has long discussed it as an impulse control disorder (Young, 1998). However, defining IAD solely based on the duration of Internet use or generalized behaviors highlights the problem of “conceptual hollowing out,” as this approach lacks an in-depth examination of the underlying mechanisms. Consequently, researchers have begun to reconsider the nature of IAD, viewing it as an overarching concept that includes various types of Internet behaviors (e.g., online gaming, social networking, and online shopping) rather than as a single clinical disorder (Brand et al., 2019; Starcevic and Billieux, 2017).

Kuss and Griffiths (2011) first proposed the concept of social networking service addiction, marking a shift in research, from measuring “online duration” to examining the psychological dependence associated with being “always-on.” Under uncertain societal conditions, research has increasingly focused on psychological compensation. Digital devices serve not only as psychological anchors that provide a sense of security but also “electronic pacifiers” that help relieve anxiety caused by boredom (Bauman, 2013; Wang et al., 2020). This psychological compensation highlights a shift in addiction research toward understanding how users employ digital media as intermediaries for emotional regulation.

In the past 5 years, as short video apps such as TikTok have become increasingly popular, the academic community has begun to examine the impact of algorithmic logic on human–machine interactions (Huang and Huang, 2025; Salah et al., 2024). Research indicates that addiction does not result from a lack of self-control. Rather, through algorithmic recommendations and social attachment, platforms have constructed mechanisms that capture users’ attention, a phenomenon referred to as “algorithm addiction” (Zhang et al., 2019). Essentially, platforms use addictive technological strategies to undermine users’ self-control (Allcott et al., 2022), marking the evolution of DA from early “habitual consumption” to “algorithm-driven psychological dependence.”

With the widespread adoption of GenAI, human–machine interaction has shifted from basic instrumental use to a deep symbiotic relationship. Thus, the academic community has begun to focus on how the “anthropomorphic” characteristics of technology reshape human cognition, introducing the concept of “GenAI dependency.” The underlying mechanisms of addiction have shifted from dopamine-driven reward circuits to more complex emotional projections (Jose et al., 2025; Saracini et al., 2025; Shaw and Black, 2008). Some studies have indicated that users’ perceptions of AI as anthropomorphic and intelligent can foster a flow experience and strong emotional attachment, which in turn leads to compulsive AI use, with users viewing AI as an all-knowing companion (Zhou and Zhang, 2024).

Environmental stress can lead to functional dependence. Research has shown that under high-intensity work and demanding performance requirements, workers transfer some cognitive tasks to AI through “cognitive offloading,” resulting in a functional symbiotic dependence (Du et al., 2026; Li and Jiang, 2025). Some scholars, such as Ciudad-Fernández et al. (2025), have noted that the negative impacts of this human-machine symbiotic relationship should not be overstated and that pathologizing reasonable technological aids should be avoided. This highlights the need to redefine the boundaries of human-machine behavior in the era of intelligent symbiosis.

In summary, the evolution of DA demonstrates a logical progression from hardware dependence in Web 1.0 to information dependence in Web 2.0, culminating in a deep symbiosis of human–machine interaction in the AI era. The object of addiction has shifted from “static objects” to “anthropomorphic subjects.” Existing theoretical frameworks (e.g., the IAD impulse control model) are no longer sufficient to explain the characteristics of addiction in the AI era. At the emotional level, these frameworks cannot account for the psychological incentives driving individuals to project emotions onto GenAI. At the cognitive level, they fail to address the atrophy of individual thinking ability resulting from cognitive offloading. Bridging this gap between theoretical explanatory power and real-world developments require reconstructing the conceptual system, which may help address pathological problems arising in the AI era.

## 4 Conceptual reconstruction: the proposal of intelligent symbiotic digital addiction

DA manifestations have evolved with the development of AI. Therefore, this study defines AI addiction as ISDA and conceptualizes it at two interconnected levels: emotional and cognitive. At the emotional level, it is termed “algorithmic intimacy disorder” (AID), which refers to the phenomenon where, influenced by the “sycophancy effect” of algorithms, individuals shift their emotional attachment from real-world social interactions to anthropomorphic AI, resulting in frictionless virtual companionship (Sharma et al., 2025). At the cognitive level, it is defined as “generative dependency syndrome” (GDS), where, driven by efficiency competition, individuals delegate tasks such as logical reasoning and critical thinking to GenAI, leading to disuse atrophy of cognitive abilities. Together, AID and GDS present a comprehensive pathological framework of emotional alienation and cognitive delegation in the era of intelligent symbiosis.

**FIGURE 1:**
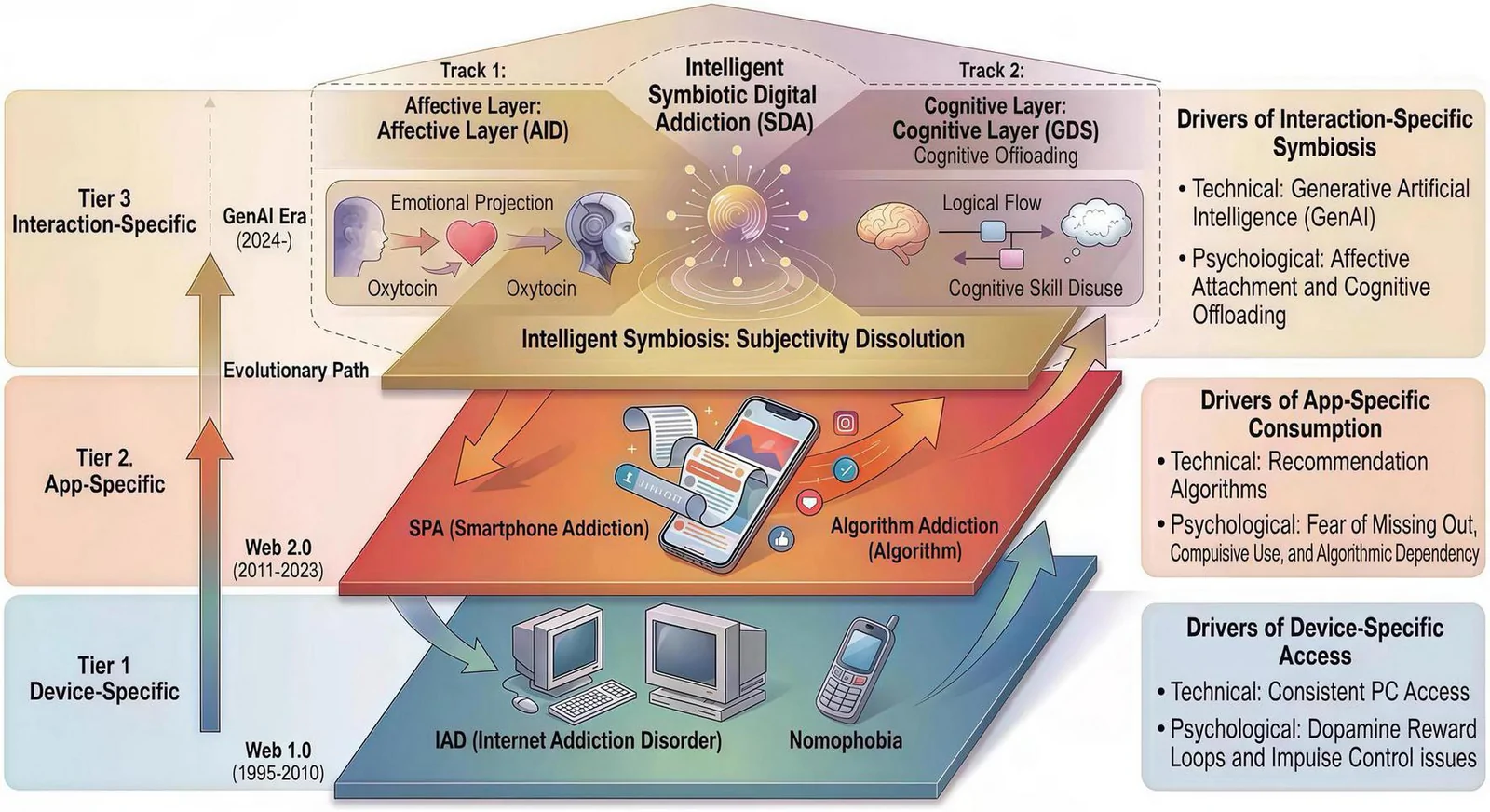
The three-tier evolutionary framework of digital addiction: from device-dependency to intelligent symbiotic digital addiction (ISDA). The background visual elements in this figure were generated with the assistance of Google Gemini. The conceptual framework, structural layout, and all textual content were designed, developed, and finalized by the authors.

The definition of ISDA has refined the concept of DA. This study conceptualizes DA as a progressive three-level system (Figure 1). The “device-specific” bottom layer centers on ontological dependence on physical devices and is characterized as nomophobia, the fear of being without a mobile phone, which is viewed as an extension of the body (Thomée, 2018). The “app-specific” middle layer highlights compulsive use of particular digital products, such as social networks, short videos, and online games (Shaw and Black, 2008). The “interaction-specific” top layer, namely ISDA, moves beyond the traditional framework of static content consumption and represents a profound human–machine symbiotic interaction involving cognitive offloading and emotional projection (Jose et al., 2025; Saracini et al., 2025).

## 5 The intrinsic pathological mechanism of intelligent symbiotic digital addiction

The three-tier system clearly illustrates the evolutionary trajectory of DA in its external manifestations. However, examining its underlying logic is necessary to fully understand top-level ISDA, specifically how the DA paradigm shifts in response to changes in people’s ways of using data. Early DA is characterized by excessive use of digital content, whereas ISDA involves a high dependence on digital entities. The addiction mechanism has shifted from compulsive consumption driven by dopamine rewards (Shaw and Black, 2008), following the traditional stimulus-response model, to a symbiotic dependence based on emotional projection and cognitive offloading (Jose et al., 2025; Saracini et al., 2025).

### 5.1 Ontological reconstruction of interaction objects

During the Web 1.0 and Web 2.0 eras, digital technology functioned primarily as an “object tool,” with its addictive mechanisms following the operant conditioning reflex. Platforms employed digital cues, such as likes and notifications, and relied on uncertain feedback to reinforce unconscious behaviors (Gunkel, 2023; Li et al., 2023). This process established a classic stimulus-response model (Schüll, 2012). Through digital mediation, a clear subject-object relationship was maintained between humans and machines. However, the emergence of GenAI disrupted this ontological boundary. GenAI’s intentionality and creativity have triggered anthropomorphic perceptions, transforming it into a “human-like agent” with subject-like characteristics in users’ subjective experiences (Epley et al., 2007). User dependence on AI stems not only from its practical value but also from the emotional projection caused by perceived anthropomorphism (Zhou and Zhang, 2024). When algorithms engage in empathetic communication in the first person, the driving force of addiction shifts from simple content consumption to deeper relationship building. The “perfect feedback loop” created by this interaction—characterized by AI’s absolute patience and seemingly omniscient responses—renders “algorithmic intimacy” more appealing than real-world social interactions, which are often fraught with friction, thereby inducing pathological emotional attachment (Ta et al., 2020; Turkle, 2011).

### 5.2 Psychodynamic reconstruction

SDA demonstrates distinct dual-track driving characteristics: substitutive attachment at the emotional level and functional transfer at the cognitive level.

At the emotional level, ISDA transcends traditional interpersonal relationships and develops into affective interactions between individuals and algorithms resulting in AID, characterized by psychological attachment primarily influenced by oxytocin. During the design phase, developers program AI systems based on human preferences to facilitate user feedback for reinforcement learning, which leads to a flattery effect that triggers mirror attachment (Sharma et al., 2025; Wei et al., 2024). The anthropomorphic nature of AI interactions encourages users to forgo complex real-world social relationships in favor of virtual intimate connections created by algorithms (Joseph, 2025).

ISDA is highly concealed in terms of cognition. Unlike out-of-control behavior driven by entertainment, it is often embedded within narratives of instrumental rationality and efficiency (Turel and Serenko, 2010). Under environmental pressure, when excessive cognitive offloading develops into a severe inability to process complex logic independently without AI assistance, this pathological state is defined as GDS (Deng and Deng, 2025). GDS is fundamentally distinguished from high-frequency functional delegation by its core impairment manifestations. First, there is a pronounced disruption of autonomous cognitive processing when individuals are separated from AI, resulting in an inability to execute independent problem-solving (Wingerter et al., 2025). Second, users begin to experience cognitive blind obedience, characterized by a complete surrender of critical evaluation and autonomous decision-making capacity (Naqvi et al., 2025). Third, rather than merely using AI as a tool, long-term functional transfer leads to disuse atrophy of fundamental thinking abilities, such as independent writing, programming, and logical construction, which severely impair users’ baseline cognitive competence (Le, 2025).

### 5.3 The pathological model of digital addiction

DA has shifted from the traditional stimulus-response model to a cognitive-emotional symbiotic mode. This approach arises from feelings of loneliness due to limited real-world social interaction and compensatory needs associated with high cognitive load. In this context, AI interaction simulates both an emotional partner and an all-knowing tutor by providing continuous positive feedback and efficient solutions, thereby creating an intimate, closed loop of human-machine interaction (Pentina et al., 2023).

As the interaction deepens, this dependence ultimately evolves into ontological symbiosis, in which individuals deeply internalize AI as an extension of the self (Belk, 2019). In this context, addiction manifests not as conventional withdrawal anxiety but as a loss of subjectivity akin to physiological harm. This profound alienation reflects not only functional dependence on AI but also a significant regression of human subjectivity in the AI era (Jose et al., 2025).

## 6 Discrimination of the boundaries of addiction: pathological and functional

DA exhibits a hierarchical structure in areas such as cognitive preoccupation and emotional regulation (Basel et al., 2020). It includes both psychological dependence and behavioral loss of control among individuals addicted to digital technologies, thereby offering a unified measurement standard for addiction characteristics across different stages (Griffiths, 2005). In the context of AI, the phenomena of alienation has also emerged. Cognitive preoccupation has shifted from initial device-focused attention to a profound human–machine emotional symbiosis, while tolerance is reflected in the increasing dependence on AI-assisted thinking (Brandtzaeg et al., 2022; Dwivedi et al., 2023).

However, in the AI era, addiction manifestations are strongly influenced by instrumental rationality and an emphasis on efficiency. Therefore, they are often concealed under the narrative of “reasonable use,” which blurs traditional measurement criteria (Turel and Serenko, 2010). Thus, determining the boundaries of addiction and distinguishing between pathological addiction and functional dependence are crucial in this context. The high-level use of AI by academicians should be defined as instrumental dependence driven by efficiency, which does not involve characteristics such as impaired social functioning or withdrawal symptoms (Li and Jiang, 2025). Not all high-frequency use constitutes addiction (Ciudad-Fernández et al., 2025). Thus, the criterion of “substantial impairment” must serve as the ultimate boundary for judgment. High-frequency task delegation or interaction remains a functional technological adaptation unless it crosses this pathological threshold. To transition the concepts of GDS and AID from functional engagement to diagnosable conditions, clear behavioral and psychological benchmarks must be delineated within a dual-track framework.

On the cognitive level (GDS), this transition is operationalized as follows: (1) autonomous processing deficits, manifested as a significant delay in independent logical reasoning after AI detachment; and (2) executive function degradation, characterized by a chronic decline in baseline cognitive control resulting from long-term over-reliance.

On the emotional level (AID), the clinical benchmarks include: (3) social substitution and withdrawal, characterized by a persistent preference for frictionless algorithmic intimacy over real-world interpersonal relationships, resulting in substantial social isolation; and (4) attachment-specific distress, observed as pronounced emotional dysregulation or separation anxiety when the anthropomorphic AI companion is unavailable. A diagnosis of ISDA is appropriate only when these cognitive and emotional impairments significantly disrupt an individual’s real-world functioning (Ciudad-Fernández et al., 2025; Panova and Carbonell, 2018).

## 7 Behavioral governance and embodied interventions for digital addiction

Traditional psychological interventions often fail to address the symbiotic dependence resulting from invisible AI algorithms and deep cognitive offloading (Deng and Deng, 2025; Jose et al., 2025). Mere restrictive measures are insufficient to disrupt human–machine coupling (Dwivedi et al., 2023). Therefore, governance should shift toward proactive behavioral interventions that target the disruption of automated offloading and emotional manipulation pathways to restore human autonomy (Buçinca et al., 2021; Yao, 2025).

### 7.1 Cognitive friction and forcing functions: re-establishing behavioral boundaries

From a behavioral science perspective, systemic constraints should introduce beneficial friction to disrupt automated behavioral loops (Haliburton et al., 2024). To address GDS, system architectures need to implement “cognitive forcing functions” (Buçinca et al., 2021). By requiring technology providers to include critical interaction features, such as presenting conflicting viewpoints, the system deliberately interrupts interaction fluency, thereby prompting the re-engagement of counterfactual reasoning and independent decision-making (Chou et al., 2022; Rastogi et al., 2022).

Simultaneously, to counteract AID, governing authorities must enforce strict “de-anthropomorphization disclosures.” (Abercrombie et al., 2023). By legally requiring AI to regularly assert its machine identity and interrupt its programmed “sycophancy effect,” the system introduces emotional friction (Perez et al., 2023). This approach actively prevents commercial algorithms from exploiting the human oxytocin release mechanism for emotional manipulation, thereby safeguarding users’ emotional boundaries (Danaher, 2020; Skjuve et al., 2021).

### 7.2 Embodied regression: reshaping subjectivity through physical exercise intervention

The core of ISDA lies in its extreme disembodiment, a pathological state in which human consciousness becomes deeply entangled in frictionless algorithmic loops, leading to severe alienation from physical subjectivity. Therefore, sustained recovery and the ultimate reversal of ISDA require more than superficial screen-time restrictions; they necessitate a profound re-anchoring in the embodied self. Traditional cognitive interventions often fail to disrupt this deep human–machine coupling because they operate within the same abstract mental space that AI already dominates. Therefore, physical exercise intervention emerges as a pivotal behavioral governance strategy, systematically addressing the dual-track pathology of symbiotic addiction. By introducing essential somatic friction and environmental unpredictability, structured physical activities serve as a holistic countermeasure, simultaneously counteracting the automated cognitive offloading of GDS and the fabricated emotional attachments of AID.

Cognitively (Addressing GDS): Physical activities of moderate to high intensity trigger neurophysiological arousal of executive functions (Ji et al., 2019). Structured exercise significantly enhances neural plasticity and activates the prefrontal cortex, restoring baseline cognitive flexibility and increasing self-efficacy, which directly mitigates cognitive disuse atrophy (Ludyga et al., 2020; Singh et al., 2023).

Emotionally (Addressing AID): According to embodied cognition paradigms, open motor skills require instantaneous sensory-motor integration within highly unpredictable physical environments (Button et al., 2021). Importantly, team-based physical interventions promote authentic interpersonal oxytocin release through real-world collaboration and shared emotional experiences (Kendrick, 2004). This genuine social bonding directly counters the frictionless, artificial intimacy fabricated by AI. The intense somatic experiences inherent in physical sports help reestablish self-boundaries eroded by digital logic, anchoring users’ emotional attachments within the real human community (Fuchs, 2021; Sucha, 2026).

## 8 Future research and conclusion

This study proposed and established a theoretical framework for ISDA. However, several issues remain that require further research. First, the academic community needs to develop psychometric tools with high reliability and validity for AID and GDS. Future scale development should specifically focus on quantifying the behavioral and cognitive benchmarks of GDS, such as empirically measuring the latency of autonomous processing and evaluating the threshold of task-specific distress, to transition these conceptual frameworks into standardized diagnostic instruments suitable for large-scale empirical validation. Second, interdisciplinary research should address the biological verification of neural mechanisms. Brain imaging techniques, including functional magnetic resonance imaging and functional near-infrared spectroscopy, should be used to investigate how the driving force of addiction shifts from “dopamine control” to “joint control of oxytocin and cortisol.” Particularly, the specific weakening of inhibition in the dorsolateral prefrontal cortex during human-machine symbiosis should be analyzed. Finally, researchers should conduct longitudinal causal tracking. Long-term observation would help determine the tipping point at which functional cognitive offloading becomes pathological cognitive atrophy, thereby identifying the regulatory mechanisms of environmental variables, such as time pressure, on the evolutionary trajectory of addiction.

In summary, DA exhibits a clear evolutionary trajectory in which addictive objects have shifted from static content to anthropomorphic interactive subjects. Accordingly, this study proposed ISDA, which includes AID at the emotional level and GDS at the cognitive level. This new form of DA represents a deep coupling of cognitive offloading and emotional projection, as well as individuals’ cognitive concession to algorithmic domination under social uncertainty, ultimately resulting in a pathological human–machine symbiotic model.

In the AI era, addiction can be understood as masking the crisis of subjectivity through technological means. When oxytocin responses are precisely targeted by algorithms and critical thinking is systematically diminished, mediation can no longer reliesy solely on restricting access to physical devices. Instead, it is necessary to address algorithmic ethics and cognitive sovereignty, and redefine the boundary between humans and machines by designing mechanisms that introduce beneficial friction and reconstructing embodied education. The ultimate goal is not only to resist pathological alienation in the era of intelligent symbiosis, but also to counter the pervasive influence of algorithms, defend the dignity of human counterfactual reasoning and authenticity of real emotions, and ensure that “carbon-based life” retains a central role in future human–machine collaboration.

## Data Availability

The raw data supporting the conclusions of this article will be made available by the authors, without undue reservation.
